# Comparative genomics of 84 *Pectobacterium* genomes reveals the variations related to a pathogenic lifestyle

**DOI:** 10.1186/s12864-018-5269-6

**Published:** 2018-12-07

**Authors:** Xiaoying Li, Yali Ma, Shuqing Liang, Yu Tian, Sanjun Yin, Sisi Xie, Hua Xie

**Affiliations:** 1 Beijing Agro-Biotechnology Research Center, Beijing Academy of Agriculture and Forestry Sciences, Beijing, 100097 People’s Republic of China; 2 Beijing Key Laboratory of Agricultural Genetic Resources and Biotechnology, Beijing, 100097 People’s Republic of China; 3 Health Time Gene Institute, Shenzhen, Guangdong 518000 People’s Republic of China

**Keywords:** *Pectobacterium carotovorum*, Comparative genomics, Genetic variation, Pathogenicity determinants, CRISPR-Cas

## Abstract

**Background:**

*Pectobacterium* spp. are necrotrophic bacterial plant pathogens of the family *Pectobacteriaceae*, responsible for a wide spectrum of diseases of important crops and ornamental plants including soft rot, blackleg, and stem wilt. *P. carotovorum* is a genetically heterogeneous species consisting of three valid subspecies, *P. carotovorum* subsp. *brasiliense* (*Pcb*), *P. carotovorum* subsp. *carotovorum* (*Pcc*), and *P. carotovorum* subsp. *odoriferum* (*Pco*).

**Results:**

Thirty-two *P. carotovorum* strains had their whole genomes sequenced, including the first complete genome of *Pco* and another circular genome of *Pcb*, as well as the high-coverage genome sequences for 30 additional strains covering *Pcc*, *Pcb*, and *Pco*. In combination with 52 other publicly available genome sequences, the comparative genomics study of *P. carotovorum* and other four closely related species *P. polaris*, *P. parmentieri*, *P. atrosepticum*, and Candidatus *P. maceratum* was conducted focusing on CRISPR-Cas defense systems and pathogenicity determinants. Our analysis identified two CRISPR-Cas types (I-F and I-E) in *Pectobacterium,* as well as another I-C type in *Dickeya* that is not found in *Pectobacterium*. The core pathogenicity factors (e.g., plant cell wall-degrading enzymes) were highly conserved, whereas some factors (e.g., flagellin, siderophores, polysaccharides, protein secretion systems, and regulatory factors) were varied among these species and/or subspecies. Notably, a novel type of T6SS as well as the sorbitol metabolizing *srl* operon was identified to be specific to *Pco* in *Pectobacterium*.

**Conclusions:**

This study not only advances the available knowledge about the genetic differentiation of individual subspecies of *P. carotovorum*, but also delineates the general genetic features of *P. carotovorum* by comparison with its four closely related species, thereby substantially enriching the extent of information now available for functional genomic investigations about *Pectobacterium*.

**Electronic supplementary material:**

The online version of this article (10.1186/s12864-018-5269-6) contains supplementary material, which is available to authorized users.

## Background

*Pectobacterium* spp. are necrotrophic bacterial plant pathogens of the family *Pectobacteriaceae*, responsible for a wide spectrum of diseases (including soft rot, blackleg, and stem wilt) of important crops and ornamental plants worldwide [1]. At the time of writing, the genus has ten species (*P. carotovorum*, *P. wasabiae*, *P. betavasculorum*, *P. cacticida*, *P. aroidearum*, *P. peruviense*, *P. atrosepticum*, *P. parmentieri*, *P. polaris*, and *Candidatus* P. maceratum), of which *P. carotovorum*, *P. atrosepticum*, and *P. parmentieri* are three soft rot species that cause the most severe economic losses [2, 3]. In contrast to *P. atrosepticum* and *P. parmentieri*, the causal agent of potato rots, *P. carotovorum* exhibits a wide host range and high heterogeneity, and is further divided into three valid subspecies: *P. carotovorum* subsp. *brasiliense* (*Pcb*), *P. carotovorum* subsp. *carotovorum* (*Pcc*), *P. carotovorum* subsp. *odoriferum* (*Pco*), as well as a the newly proposed but as yet not validly published subspecies *P. carotovorum* subsp. *actinidiae* (*Pca*) [4–6]. In addition, some strains previously known as *P. carotovorum* has been separated from *P. carotovorum* recently and have been proposed as four new species: *P. aroidearum*, *P. peruviense*, *P. polaris* and *Candidatus* P. maceratum [7–10].

*Pectobacterium* spp. possess a great number of pathogenicity determinants that contribute to the ability to cause maceration-associated diseases [4]. Most of them are secreted and injected into host cells through one or a combination of the six known types of bacterial protein secretion systems: T1SS-T6SS [11]. Recent studies on CRISPR-Cas systems, which function as a type of microbial immune system, may also influence *Pectobacterium* pathogenicity [12]. We focus our studies on genome-level variation in known virulence factors such as Plant Cell Wall Degrading Enzymes (PCWDEs), secretion systems, flagellin, siderophores, and polysaccharides, as well as CRISPR-Cas systems, in genus *Pectobacterium,* and especially in the heterogeneous species *P. carotovorum* in order to improve our understanding of the genetic basis for pathogenicity in general.

Genome sequencing has provided new insights into the lifestyle of *Pectobacterium*. *P. atrosepticum* SCRI1043 (accession: BX950851) was the first soft rot pathogen sequenced in 2004 [11]. By July 2018, many genome data have been released successively of which 16 more *Pectobacterium* complete genome sequences have become publicly available: *P. atrosepticum* 21A (accession: CP009125) [13], *P. atrosepticum* JG10–08 (accession: CP007744), *P. wasabiae* CFBP3304 (accession: CP015750), *P. aroidearum* PC1 (accession: CP001657), *P. parmentieri* WPP163 (accession: CP001790), *P. parmentieri* SCC3193 (accession: CP003415) [14], *P. parmentieri* RNS08.42.1A (accession: CP015749) [3], *P. carotovorum* 3–2 (accession: CP024842) and PCC21 (accession: CP003776) [15], *Candidatus* P. maceratum SCC1 (accession: CP021894) [16], *P. polaris* NIBIO1392 (accession: CP017482) and NIBIO1006 (accession: CP017481) [9], *Pcb* SX309 (accession: CP020350) and BZA12 (accession: CP024780) [17], as well as two new sequenced genomes *Pcb* BC1 (CP009769), *Pco* BCS7 (accession: CP009678) in this study (Additional file 1: Table S1). Previous genomic studies have compared *Pectobacterium* with other genera [18–20], and we are aware of two studies that examined *Pectobacterium* species/subspecies differences among *P. atrosepticum*, *Pcc*, and *Pcb* [21], and another comparing *P. parmentieri* and *P. atrosepticum* [22]. However, no reports have as yet compared the genomes of all of the subspecies of *P. carotovorum*.

The present study provides an important resource for functional genomics studies of the heterogeneous *P. carotovorum*. A total of 32 *P. carotovorum* strains including 14 *Pcb*, 5 *Pcc*, and 13 *Pco* strains were collected from 3 economically important Chinese vegetables (Chinese cabbage, Bok choy, and celery) (Additional file 1: Table S1 and Additional file 2: Figure S1), and these were characterized by conducting full genome sequencing and extensive phenotypic analyses. In addition, we conducted comparative genomics based on the 32 newly sequenced sequences and 52 additional genomes from *P. carotovorum*, *P. polaris*, *Candidatus* P. maceratum, *P. atrosepticum*, and *P. parmentieri* available from GenBank with three complete *Dickeya* genomes (*D. zeae* Ech586, *D. paradisiaca* Ech703, and *D. dadantii* 3937) as the reference. Our results not only represent progress in the knowledge regarding the genetic differentiation of individual subspecies of *P. carotovorum*, but also delineate the general genetic features of *P. carotovorum* by comparison with that of other four closely related species in following aspects: phylogenetic analysis, general genomic properties, genome similarity, pan-genome, CRISPR-Cas systems, pathogenicity-related genes, and TNSSs. The results showed the *srl* operon/T6SSc is apparently specific to *Pco* subspecies, and we therefore further studied the distribution and the similarity of *srl* operon/T6SSc in the *Pectobacterium* genus and even in the *Enterobacterales* order by using an expanding genome data set. This study represents a major step towards filling in our understanding about genomic differentiation in pathogenicity-related genes and pathways of the economically important *Pectobacterium* soft rot pathogens.

## Result

### Phenotypic and genomic features of 32 Chinese *Pectobacterium carotovorum* isolates

Thirty-two Chinese soft rot isolates including 14 *Pcb*, 5 *Pcc*, and 13 *Pco* strains were collected from 3 economically important Chinese vegetables (Chinese cabbage, bok choy, and celery) afflicted with bacterial soft rot disease (Additional file 2: Figure S1). All isolates tested caused soft rot lesions at the locations that were inoculated onto Chinese cabbage petioles (Additional file 1: Table S1). The extent of the decay caused by the *Pcb* strains (mean diameter of decay in Chinese cabbage = 3.29 cm) was, on average, less substantial than the decay caused by the *Pcc* or *Pco* strains (mean diameter of decay = 3.62 and 3.72 cm, respectively); however, consider that the *Pcb* strains exhibited a very wide range of values *Pcb* (0.24~4.06 cm), *Pcc* (range 3.13~3.83 cm), and *Pco* (range 1.19~4.20 cm). All tested strains were able to grow at 37 °C and were positive for utilization of cellobiose, sucrose, α-D-glucose, D-fructose, D-mannitol, and glycerol. They were unable to utilize propionic acid, L-alanine, D-maltose, D-serine, tween 40, and L-histidine. However, all tested *Pco* strains produced acid from D-sorbitol, palatinose, α-methylglucoside, and utilized D-arabitol whereas the *Pcc* and *Pcb* strains could not (Additional file 1: Table S1-S2)*.*

**Fig. 1:**
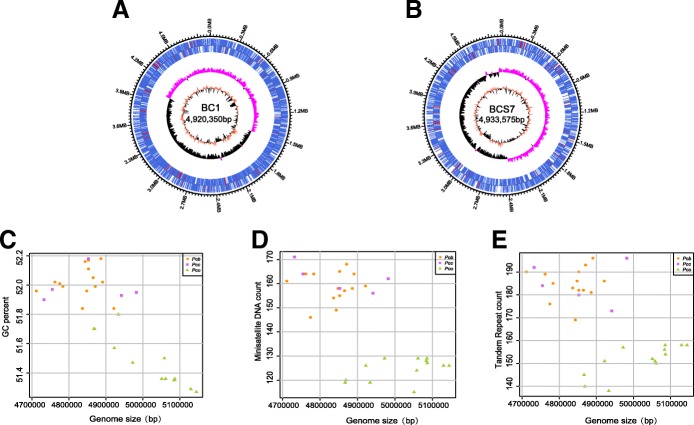
Genomic features of the 32 newly sequenced *Pectobacterium carotovorum* strains. **a** Circular chromosome map of *Pcb* strain BC1. **b** *Pco* strain BCS7. The distribution of genes is shown on the two outer rings. The next circle indicates the GC-skew values (pink: the GC-skew values > the average; black: the GC-skew values < the average) and the central circle shows the GC content (salmon: the GC content> the average; black: the GC content < the average). Two-dimensional plots showing relationships between genome size and GC content (**c**), minisatellite DNA count (**d**), and TR count (**e**) for the 32 newly sequenced *P. carotovorum* strains. The salmon dots represent the 14 *Pcb* strains, the pink squares represent the 5 *Pcc* strains, and the light green triangles represent the 13 *Pco* strains

Subsequently, to examine their genomic variation that may underly phenotypic differences, we sequenced the 32 *P. carotovorum* isolates to an average depth of 100-fold coverage. The genomes of both *Pcb* BC1 and *Pco* BCS7 (Fig. 1a and b) consist of a single circular chromosome of 4,920,350 bp and 4,933,575 bp, GC content of 51.84 and 51.75%, 4868 and 3855 predicted CDS sequences, respectively, and both possessing 7 rRNA operons, and 77 tRNA genes. The sizes of the draft genomes for 30 additional *P. carotovorum* strains ranged from 4,710,507 to 5,144,582 bp with GC% content ranging from 50.97 to 52.18% (Additional file 1: Table S3). No apparent autonomous plasmids were identified among these strains. We found that several genomic features that distinguish these Chinese *Pco* strains from others: the genomes of these *Pco* strains are the largest in size, have the lowest GC content, and have the least number of both tandem repeats and minisatellite DNA sequences compared with these Chinese *Pcc* and *Pcb* strains (Fig. 1c, d, and e).

### Comparative genomics analysis of *Pectobacterium carotovorum* and other closely related species

To enrich the extent of information available for functional genomic investigations about *P. carotovorum*, we conducted comparative genomics analyses of individual subspecies of *P. carotovorum* and compared *P. carotovorum* genetic features with those of *P. atrosepticum*, *P. parmentieri*, *P. polaris*, *Candidatus* P. maceratum. A total of 84 *Pectobacterium* genomes including the 32 newly sequenced *P. carotovorum* genomes and the other available 52 *Pectobacterium* genomes downloaded from GenBank in July 2018 were used in this analysis (Additional file 1: Table S3).

#### Genomics features for distinguishing closely related species

Generally, we found three major genomic features of these 84 *Pectobacterium* genomes seemed to have regularity with their classification of species and subspecies, including GC content and the numbers of both long terminal repeats and minisatellite DNA sequences (Additional file 1: Table S4)*.* The differing GC content of closely related species has been considered as a useful means of discrimination for a long time [23]. At the subspecies level, *Pco* and *Pca* had lower GC content (51.27–51.70%) than the other two subspecies (*Pcc* and *Pcb*) (51.84–52.18%; except *Pcc* Y16 (50.97%) and *Pcb* CFIA1033 (51.25%)). At the species level, *P. carotovorum* had a similar GC content with *P. polaris* and *Candidatus* P. maceratum, which was higher than *P. atrosepticum* (50.87–51.15%), whereas *P. parmentieri* (50.37–50.59%) had the lowest GC content among these four species. Overall, these *Pectobacterium* spp. had a lower GC content (50.37–52.18%) in comparison with the three *Dickeya* strains (53.63–56.29%). There were also fewer long terminal repeats in *P. carotovorum*, *P. polaris*, and *Candidatus* P. maceratum (56–122; except *Pcc* WPP14 (134), *Pcb* PBR1692^T^ (137), and *Pca* ICMP 19971 (126)) than in the other two species *P. atrosepticum* and *P. parmentieri* (125–157). For minisatellite DNA sequences, *Pca* had the lowest number (84–88) among these subspecies and species, and the number of *Pco* (115–129; except NCPPB3841 (143)) was less than that of *Pcc*/*Pcb* (131–172) at the subspecies level. In addition, *P. parmentieri* had fewer such sequences (94–100) than *P. atrosepticum* (124–141), which was far fewer than that in *Candidatus* P. maceratum or *P. polaris* (148–208) at the species level.

#### Genome similarity of Pectobacterium carotovorum and other species as well as that of P. carotovorum subspecies

**Fig. 2:**
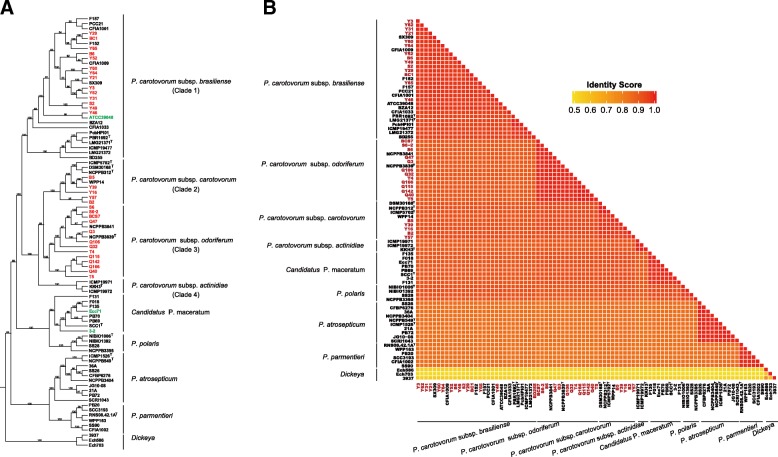
Taxonomy and Identity Score analysis of the 84 *Pectobacterium* genomes. **a** Maximum likelihood tree based on SNP data for the 84 *Pectobacterium* genomes including 56 *P. carotovorum* (that is 9 *Pcc*, 29 *Pcb*, 15 *Pco*, and 3 *Pca* genomes), 6 *P. parmentieri*, 10 *P. atrosepticum*, 4 *P. polaris*, and 8 *Candidatus* P. maceratum genomes, with 3 *Dickeya* genomes (*D. paradisiaca* Ech 703, *D. zeae* Ech586, and *D. dadantii* 3937) as the outgroup. The tree was generated with 1000 bootstrap replicates. The strains with red letters are the newly sequenced *Pectobacterium* strains in this study and the three *P. carotovorum* strains with green letters are the apparently misnamed strains detailed in the text. **b** Heat map (color code in the ramp, above) of identity scores based on pairwise comparison of these 84 *Pectobacterium* genomes and 3 *Dickeya* genomes

The topologies of maximum likelihood tree based on the huge SNP data from the 84 *Pectobacterium* genomes revealed that the four closely related species formed two major well-resolved groups (Fig. 2a). One group consisted of species *P. carotovorum*, *P. polaris*, and *Candidatus* P. maceratum, while the species *P. parmentieri* and *P. atrosepticum* formed the other group. Strains of *P. carotovorum* were further clustered into four distinct clades: clade 1, for *Pcb* strains and another one *Pcc* strain (ATCC 39048); clade 2, for *Pcc* strains; clade 3, for *Pco* strains; and clade 4, for *Pca* strains. Clade 1 was split further as follows: one subclade contained the type strain *Pcb* LMG 21371^T^ as well as another five published *Pcb* strains from Brazil, New Zealand, and South Africa, and the other subclade harbored all the strains of the subspecies isolated from China, Russia, Canada, Korea as well as one *Pcc* strain from USA. This indicates that significant genetic diversity exists among different *Pcb* strains related to the origins, and the *Pcc* strain ATCC 39048 appear to be misnamed. It was also showed that a close relationship exists between *Pcc* and *Pco*, as they were gathered into a single large group that was separated from the *Pcb* strains. The Chinese *Pco* strains isolated from celery and Chinese cabbage were clustered with the type strain of *Pco* NCPPB 3839^T^ and the other *Pco* strain isolated from chicory. In addition, two strains *P. carotovorum* 3–2 and *Pcc* Ecc71 were clustered with the other published *Candidatus* P. maceratum strains suggesting an erroneous classification.

To characterize genomic similarity within each of subspecies/species, identity scores (IS) (Fig. 2b) were calculated based on the detected 52,630 SNPs among these tested strains. At the species level, we found *P. carotovorum* was most similar to *Candidatus* P. maceratum (90.28–93.49%) and least similar to *P. atrosepticum* (83.62–84.94%) and *P. parmentieri* (83.28–85.76%). Within subspecies *carotovorum*, *Pcb* was more closely related to *Pcc* than to *Pco* and *Pca*, since *Pcb* strains had the higher IS values with *Pcc* strains (91.25–93.39%) than with *Pco* (89.65–90.63%) and *Pca* strains (89.01–89.78%). A close relationship was also observed between *Pcc* and *Pco* with higher similarity (92.79–93.79%) than that of any other two subspecies. *Pca* strains also had high similarity with *Pcc* (91.31–91.80%) and *Pco* (90.50–90.68%) strains but had low similarity with *Pcb* (89.01–89.78%). It is noteworthy that the similarity of three Korean *Pca* strains (KKH3^T^, ICMP 19971, and ICMP 19972) isolated from kiwi fruit [6, 24] were 100% amongst each other.

#### Pan-genome analysis identified a srl operon specific to Pco

**Fig. 3:**
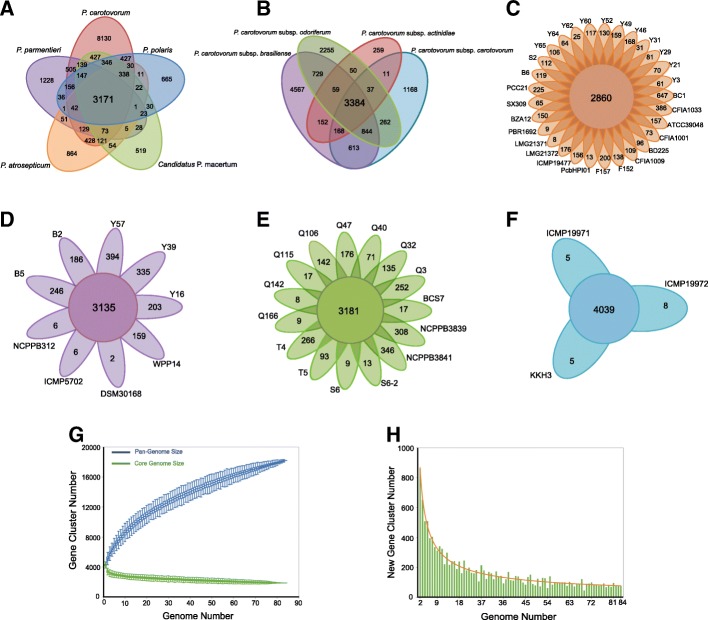
Pan- and core-genome analyses of the 84 *Pectobacterium* genomes. The number of genes in the pan-genomes and core-genomes, and the unique genes among the genomes were classified using CD-HIT, with a threshold of 70% pairwise identity and a 0.6 length difference cut-off in amino acid sequences. **a** Venn diagram of *Pectobacterium* genus for 56 *P. carotovorum*, 4 *P. polaris*, 8 *Candidatus* P. maceratum, 10 *P. atrosepticum*, and 6 *P. parmentieri* genomes. **b** Venn diagram of *P. carotovorum* species for 29 *Pcb,* 9 *Pcc*, 15 *Pco*, and 3 *Pca* genomes. The core orthologous genes are not the genes that are shared by all of the tested genomes of a given group; rather these are the genes that were present in a minimum of one genome from each of the targeted subgroup. **c-f** Flower plots of each of four subspecies for 29 *Pcb,* 9 *Pcc*, 15 *Pco*, and 3 *Pca* genomes, respectively. The figures of 84 *Pectobacterium* genomes in (**g**) and (**h**) were produced using PanGP. **g** Plot in which the blue line shows the impact on the number clusters for the pan-genome resulting from the addition (simulated, randomized) of further genomes to the analysis, with the asymptotic value of *y* = *Ax*^*B*^ + *C*; the green line shows how such addition influenced the number of core genes, with the asymptotic value of *y* = *Ae*^*Bx*^ + *C*. **h** Histogram showing the increases in the number of new (unique) genes as the number of genomes was increased in our pan-genome analysis. The horizontal dashed line (orange) indicates the asymptotic value with the function of *y* = *Ax*^*B*^

We performed a pan-genome analysis of *Pectobacterium* based on 36,634 putative protein-encoding sequences from the 84 *Pectobacterium* genomes sequences. The *Pectobacterium* pan-genome can be subdivided into three categories: (i) the core genome (that is, the set of genes present in the genome of at least one strain of each subgroup; not a gene that is shared by all strains), (ii) the accessory genome (the set of genes present in some but not all subgroups), and (iii) the unique genome (genes that are unique to a single subgroup) (Fig. 3). The core genome of *Pectobacterium* consists of 3171 (17.47%) out of a total of 18,147 orthologous genes. OrthoMCL comparative analysis of shared genes revealed a higher number of genes commonly present in *P. carotovorum*-*Candidatus* P. maceratum (4762), and *P. carotovorum*-*P. polaris* (4657), than that in common between *P. carotovorum-P. atrosepticum* (4332), and *P. carotovorum*-*P. parmentieri* (4362), supporting our observations from the abovementioned IS analysis that showed a closer genetic relationship between *P. carotovorum* and *Candidatus* P. maceratum than with *P. polaris*, *P. atrosepticum,* or *P. parmentieri* (Fig. 3a). The core orthologous genes and subspecies-specific unique genes within a given *P. carotovorum* species were examined in 9 *Pcc*, 15 *Pco*, 29 *Pcb*, and 3 *Pca* strains (Fig. 3b). A total of 14,558 homologs were identified. Of these, 3384 orthologs (23.24%) were identified as the *P. carotovorum* core genome. A total of 5016 genes were shared between *Pco* and *Pcb*, while *Pcc* shared fewer orthologs with *Pcb* (5009) and *Pco* (4527) and *Pca* shared few orthologs with the other three subspecies: *Pcb* (3763), *Pcc* (3600), and *Pco* (3530), highlighting the relatively higher extent of genetic differentiation of *Pca* compared with the other three subspecies. The differentiation was also presented between different strains within the same subspecies in *P. carotovorum* (Fig. 3c, d, e, and f).

Additionally, the size of the pan-genome and its increase in size upon addition of new strains can be used to predict the future rate of discovering novel genes in a species (Fig. 3g and h): the pan-genome curve fit a Heaps’ law pattern (exponent > 0) and was considered “open”, which is typical of species colonizing multiple environments and having several means of exchanging genetic material [25]. This analysis highlights the large genomic diversity of *Pectobacterium* species and suggests that these species can very likely adapt to a wide variety of hosts. The COG (Clustering of orthologous groups) analysis of unique genes of *P. atrosepticum*, *P. parmentieri*, *P. polaris*, *Candidatus* P. maceratum, as well as the subspecies of *P. carotovorum* showed that the *srl* operon (BCS7_10600-BCS7_10625) that is known to facilitate sorbitol utilization [26] was found only in the *Pco* strains, suggesting that this *srl* operon may be specific to *Pco* subspecies. The specific distribution of this operon in *Pectobacterium* genus were future confirmed in a subsequently expanded genome analysis using all of the available genomes from GenBank. These findings highlight substantial divergence among *Pectobacterium* species and subspecies. Seeking to better understand the genetic basis of pathogenicity and host specificity, our next analyses specifically compared the similarity and divergence of the genes encoding CRISPR-Cas, virulence determinants, regulatory mechanism, and protein secretion systems.

#### The occurrence of I-E type CRISPR-Cas systems in Pectobacterium

**Fig. 4:**
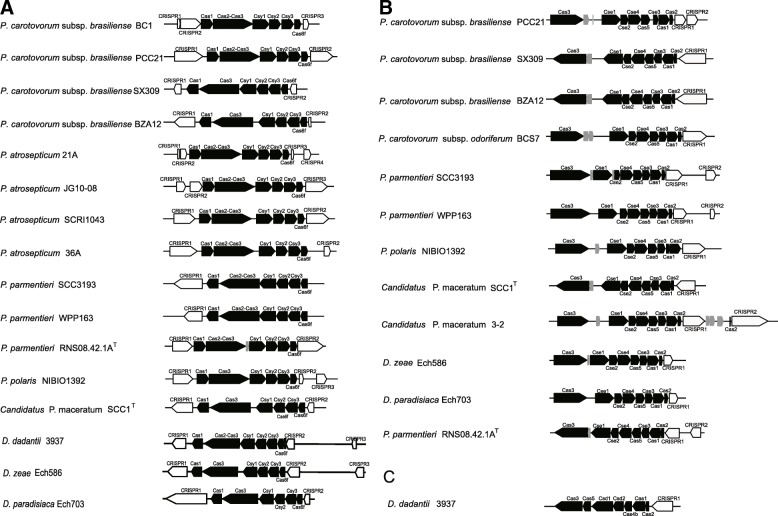
Representative CRISPR-Cas systems types identified in this study. All of the 84 *Pectobacterium* genomes and 3 out-group *Dickeya* genomes were identified their CRISPR-Cas systems in this study. The strains with complete genomes were selected to show their CRISPR-Cas systems architectures here, and the detailed information of CRISPR-Cas systems in all of the tested genomes were given in Additional file 1: Table S5. The diagram features in black represented the genes encoding known Cas proteins, features in grey represented the genes predicted to encode hypothetical proteins of unknown function, and features in white represent CRISPR sequences. **a** I-F type of CRISPR-Cas, **b** I-E type of CRISPR-Cas, and **c** I-C type CRISPR-Cas systems in these complete genomes are shown

We investigated the CRISPR-Cas bacterial defense systems looking for genome-level variations that may influence the pathogenicity of *Pectobacterium* [12]. To date, surveys of the CRISPR-Cas systems in *Pectobacterium* have only been performed for *P. atrosepticum* SCRI1043, in which a single I-F type system was identified [27]. In the present study, we identified I-F, I-E type systems among these available *Pectobacterium* genomes, with seven exceptions among the *Pectobacterium* strains (*Pcb* LMG 21372, ICMP 19477, CFIA1001, and Y46; *Pco* NCPPB3841, NCPPB 3839^T^, and Q3; *P. polaris* NIBIO1006^T^) that lacked any CRISPR-Cas system (Table 1). The distribution of these systems varied among *Pectobacterium* species and subspecies: most of the *P. carotovorum*, *P. polaris*, and *Candidatus* P. maceratum strains contained I-F or I-E systems, and some strains carried both types (28% *Pcb*, 33% *Pcc*, 13% *Pco*, 25% *P. polaris*, and 50% *Candidatus* P. maceratum); all of the tested *Pca* and *P. atrosepticum* strains have a single I-F type, while both *P. parmentieri* strains have both the I-F and I-E types (Table 1). Moreover, our analysis identified CRISPR-Cas types (I-F, I-E and I-C) in reference *Dickeya* genomes*,* which have not been reported previously, as well as a I-C type in *D. dadantii* 3937 that is not found in *Pectobacterium*. The CRISPR-Cas systems from complete genomes of 84 *Pectobacterium* genomes, as well as three *Dickeya* genomes, are shown in Fig. 4, and the CRISPR-Cas systems of all the tested genomes in this study are shown in Additional file 1: Table S5.

**Table 1:** The identified types of CRISPR-Cas system in the 84 *Pectobacterium* genomes

Species/Subspecies	Strain	Number of each CRISPR-Cas system type		
		I-F	I-E	I-C
*P. carotovorum* subsp. *brasiliense*	PBR1692^T^ = LMG 21371^T^, **BC1,** B6, Y3, Y29, Y31, Y60, Y62, Y64, CFIA1009, CFIA1033, PcbHPI01, BD255, ATCC 39048, F157, F152	1	0	0
	**PCC21**, **SX309**, **BZA12**, Y21, Y49, Y52, Y65, S2	1	1	0
	LMG 21372, ICMP 19477, CFIA1001, Y46	0	0	0
*P. carotovorum* subsp. *carotovorum*	Y57	1	0	0
	DSM 30168^T^ = NCPPB 312^T^ = ICMP 5702^T^, B2, Y16	0	1	0
	B5, Y39, WPP14	1	1	0
*P. carotovorum* subsp. *odoriferum*	Q40, Q106, Q115, Q142, Q166, T4, T5	1	0	0
	**BCS7**, S6, S6–2,	0	1	0
	Q32, Q47	1	1	0
	NCPPB 3839^T^, NCPPB3841, Q3	0	0	0
*P. carotovorum* subsp. *actinidiae*	ICMP 19972, ICMP 19971, KKH3^T^	1	0	0
*Candidatus* P. maceratum	F018, F135, Ecc71	1	0	0
	**3–2**	0	1	0
	**SCC1**^T^, F131, PB69, PB70	1	1	0
*P. polaris*	NCPPB 3395, SS28	0	1	0
	**NIBIO1392**	1	1	0
	**NIBIO1006** ^T^	0	0	0
*P. atrosepticum*	ICMP 1526^T^ = NCPPB 549^T^, **36A**, **21A**, CFBP 6276, NCPPB 3404, PB72, SS26, **JG10–08**, **SCRI1043**	1	0	0
*P. parmentieri*	**RNS08.42.1A**^T^, **SCC3193**, **WPP163**, SS90, CFIA1002, PB20	1	1	0
*D. dadantii*	**3937**	1	0	1
*D. zeae*	**Ech586**	1	1	0
*D. paradisiaca*	**Ech703**	1	1	0

The strains with bold black font are strains with completely sequenced genomes, whose CRISPR-Cas systems architectures are shown in Fig. 4, and the CRISPR-Cas systems of all strains in this study are shown in Additional file 1: Table S5

#### Inconsistently distributed pathogenicity-related genes among Pectobacterium species and subspecies

To obtain a more comprehensive view of the pathogenicity factors present among *Pectobacterium* spp., we mined the 84 *Pectobacterium* genomes to identify previously known pathogenicity determinants, including PCWDEs, polysaccharides, iron uptake proteins, pili, and regulatory systems. Many pathogenicity determinants (e.g., most PCWDEs, enzymes for synthesis of enterobacterial common antigen (ECA) and the 3-hydroxy-2-butanone pathway) (Additional file 1: Table S6 and Table S6–1) are commonly existed among strains due to their importance in ensuring disease development caused by *Pectobacterium* spp. However, we also found inconsistent distributions of several single genes or large DNA fragments/clusters that possibly contribute to pathogenicity in variable or context-dependent scenarios for several species and subspecies (Table 2). All of the *P. carotovorum*, *P. polaris*, and *Candidatus* P. maceratum strains, but none of *P. atrosepticum*, and *P. parmentieri* strains, had the gene *fhaB* (BASYS01817) whose homolog encodes the filamentous hemagglutinin that influences virulence by human pathogens [28]. No regular distribution was observed on the two clusters (*waa* and *rfb*) encoding lipopolysaccharide (LPS) biosynthesis proteins present in *P. carotovorum* strains. The variation of LPS-related gene composition within the strains in this study likely reflects adaptations by *Pectobacterium* in the LPS-mediated plant-host interactions at the cell surface [22]. Notably*,* all of the *P. parmentieri* strains examined in this study lacked three PCWDEs genes (*pel*, *pelA*, and *pgl*) and a transport system for a ferric citrate cluster (*fecABCDE*), while possessing unique genes for siderophore biosynthesis and iron transport such as the genes *iucABCD* [29].

**Table 2:** The distribution of genes/clusters related to virulence among 84 *Pectobacterium* genomes

Gene/cluster	Locus tag	*Pectobacterium*								Reference *Dickeya* strains^a^		
		*Pc* (*n* = 56)				*Pa* (*n* = 10)	*Pp* (*n* = 6)	*CPm* (*n* = 8)	*Ppo* (*n* = 4)	*D. zeae* Ech 586	*D. dadanti* 3937	*D. paradisiaca* Ech703
		*Pcb* (*n* = 29)	*Pcc* (*n* = 9)	*Pco* (*n* = 15)	*Pca* (*n* = 3)							
Cellulases (PCWDEs)												
*celV/celB*	BCS7_11780/BCS7_07535	29	9	15	3	10	6	8	4	–	–	–
putative cellulase	BCS7_10680	29	9	15	3	10	6	8	4	Dd586_2061	Dda3937_00081	–
Oligogalacturonide (PCWDEs)												
*ogl*	BCS7_09745	29	9	15	3	10	6	8	4	Dd586_1940	Dda3937_03686	Dd703_2056
Pectate_lyase (PCWDEs)												
*pelX/pelB/pelW*	BCS7_21380/BCS7_11020/BCS7_09865	29	9	15	3	10	6	8	4	Dd586_4161/Dd586_3789/Dd586_1962	Dda3937_00143/Dda3937_04192/Dda3937_03361	Dd703_3996/Dd703_0352/Dd703_2033
*pel*	BCS7_14745	29	9	15	3	10	0	8	4	–	–	–
*pelA*	BCS7_11155	29	9	15	3	10	0	8	3	Dd586_3083	Dda3937_03370	Dd703_2763
*pelL/pelZ*	BCS7_08875/BCS7_19275	29	9	15	3	10	6	8	4	Dd586_1488/Dd586_3790	Dda3937_02794/Dda3937_04191	Dd703_1887/Dd703_0351
*pel1*/*pel2*/*pel3*	BCS7_19260-BCS7_19270	29	9	15	3	10	6	8	4	–	–	–
*pelI*	BCS7_05195	29	9	15	3	10	6	8	4	Dd586_2937	Dda3937_00058	–
Pectin acetylesterase (PCWDEs)												
*paeX/paeY*	BCS7_09835/BCS7_15435	29	9	15	3	10	6	8	4	Dd586_1956/Dd586_3086	Dda3937_03363/Dda3937_03373	Dd703_2039/Dd703_2765
Pectin lyase												
*pnl*	BCS7_06950	29	9	15	3	10	6	8	4	–	Dda3937_03551	Dd703_3378
Pectin methylesterase (PCWDEs)												
*pemA*	BCS7_15440	29	9	15	3	10	6	8	4	Dd586_3087	Dda3937_03374	Dd703_2766
*pemB*	BCS7_00330	29	9	15	3	10	6	8	4	–	Dda3937_03435	–
Polygalacturonase (PCWDEs)												
*pgl*	BCS7_17010	29	9	15	3	9	0	8	4	Dd586_3319	Dda3937_00206	Dd703_0972
*pehX*	BCS7_14740	29	9	15	3	10	6	8	4	Dd586_3904	Dda3937_00261	Dd703_0200
*pehN*	BCS7_05675	29	9	15	3	10	6	8	4	–	Dda3937_04155	–
*pehA*	BCS7_05200	29	9	15	3	10	6	8	4	–	–	–
Rhamnogalacturonate lyase (PCWDEs)												
*rhiE*	BCS7_03460	29	9	15	3	10	6	7	4	Dd586_2097	Dda3937_01465	–
Pilus assembly proteins												
*pilA*	BCS7_01135	1	0	15	0	0	0	0	0	–	–	–
*cpaABCDEF*	BCS7_01140-BCS7_01165	1	0	15	0	0	0	0	0	–	–	–
*tadBCDE*	BCS7_01170-BCS7_01185	1	0	15	0	0	0	0	0	–	–	–
*-*	BCS7_01190-BCS7_01230	1	0	15	0	0	0	0	0	–	–	–
*papA*	BASYS00091	28	9	0	3	10	1	8	3	–	–	–
*papC*	BASYS00090	28	9	0	3	10	5	8	3	–	–	–
*papD*	BASYS00089	28	9	0	3	10	6	8	3	–	–	–
*fhaB*	BASYS01817	29	9	15	3	0	0	8	4	–	–	–
Siderophore synthetase												
*entB*	BASYS04531	29	9	15	3	10	0	8	4	Dd586_2819	Dda3937_02432	Dd703_1488
*foxA*	BASYS03462	29	9	15	3	10	0	8	4	–	–	Dd703_0458
*iucABCD*	W5S_0838-W5S_0841	0	0	0	0	0	6	0	0	–	–	–
*-*	W5S_0842	0	0	0	0	10	6	0	0	–	–	–
*-/entS/ fepD/fepG*	W5S_3869-W5S_3872	0	0	0	0	0	6	0	0	Dd586_2808-Dd586_2811	Dda3937_03032-Dda3937_03035	Dd703_1499-Dd703_1496
*fepC*	W5S_3873	29	9	15	3	10	6	8	4	Dd586_2812	Dda3937_03036	Dd703_1495
*-/mbtH/−/−*	W5S_3874-W5S_3877	0	0	0	0	0	6	0	0	Dd586_2813-Dd586_2816	Dda3937_03038-Dda3937_03042	Dd703_1494-Dd703_1491
*-*	W5S_3878	0	0	0	0	0	6	0	0	–	–	–
*fyuA/−*	BASYS00782/BASYS00783	28	9	0	3	0	0	8	3	–	–	–
*-*	BASYS00784	28	5	0	3	0	0	1	0	–	–	–
*pchR/msbA*	BASYS00785/BASYS00786	28	9	0	3	0	0	8	3	–	–	–
*ygaD*	BASYS00787	28	9	0	3	0	0	8	4	–	–	–
*fecABCDE*	BASYS00244-BASYS00240	27	4	0	3	10	0	8	3	–	–	–
*nosA*	BCS7_07445	0	0	15	3	10	6	8	0	–	–	–
Synthesis of LPS												
*rfaD/waaF*^b^*/waaC*	W5S_4520-W5S_4523	29	9	15	3	10	6	8	4	Dd586_0164-Dd586_0162	Dda3937_02041-Dda3937_02039	Dd703_0173-Dd703_0171
*waaL1*	W5S_4524	18	6	15	3	10	6	2	2	–	–	–
*waaL2*	W5S_4526	0	0	0	0	7	2	0	0	–	–	–
*waaQ/waaG*	W5S_4527/W5S_4528	18	6	15	3	10	6	2	2	–	–	–
*waaI*	W5S_4529	18	6	10	3	10	6	2	2	–	–	–
*waaJ*	W5S_4530	3	3	3	0	7	2	1	0	–	–	–
*-*	W5S_4532	7	0	0	0	0	2	0	0	–	–	–
*waaX*	W5S_4533	0	0	0	0	7	2	0	0	–	–	–
*kdtX*	W5S_4534	26	6	5	3	7	2	7	3	Dd586_0155	Dda3937_02032	Dd703_0164
*-*	W5S_4535	6	4	5	0	7	2	1	0	–	–	–
*kdtA/kdtB*	W5S_4536/W5S_4537	29	9	15	3	10	6	8	4	Dd586_0156/Dd586_0154	Dda3937_02033/Dda3937_02031	Dd703_0165/Dd703_0163
*rfbP*	ECA1420	4	2	8	0	10	2	0	1	–	–	–
*rfbI/rfbF/rfbG/rfbH*	ECA1421-ECA1424	0	0	1	0	9	0	1	0	–	–	–
*hpcH/−/−/nahO/nahM/−/rfbX/−*	ECA1425-ECA1432	0	0	0	0	9	0	0	0	–	–	–
*-*	ECA1433	0	0	0	0	9	0	0	0	Dd586_0507	–	–
*−/rfc/rfbU*	ECA1434-ECA1436	0	0	0	0	9	0	0	0	–	–	–
*rfbN*	ECA1437	1	2	6	0	9	0	0	1	Dd586_1241	Dda3937_03925	–
*rfbM*	ECA1438	15	3	3	0	9	0	6	1	Dd586_1250	Dda3937_00442	Dd703_3281
*rfbK*	ECA1439	12	3	2	0	9	0	6	1	Dd586_1251	Dda3937_00443	Dd703_3280
*rfbD/rfbC*	ECA1440/ECA1441	18	6	15	3	10	6	2	3	Dd586_1246/Dd586_1245	Dda3937_03921/Dda3937_03922	Dd703_3287/Dd703_3288
*rfbA*	ECA1442	28	9	15	3	10	6	8	4	Dd586_1244	Dda3937_03923	Dd703_3286
Synthesis of ECA												
*wecG/wzyE/wecF/wzxE/rffA/rffC/rffG/wecC/wecB*	BASYS03691-BASYS03699	29	9	15	3	10	6	8	4	Dd586_3890-Dd586_3897	Dda3937_00277-Dda3937_00270	Dd703_0212-Dd703_0205
*wecA/wzzE*	BASYS03700/BASYS03701	29	9	15	3	10	6	8	4	Dd586_3898/Dd586_3899	Dda3937_00269/Dda3937_00268	–
Metabolic regulatory protein												
*metJ*	BCS7_20315	29	9	15	3	9	6	8	4	Dd586_3934	Dda3937_03887	Dd703_3792
Necrosis-inducing virulence protein												
*nip*	BCS7_14620	29	9	15	3	10	6	8	4	Dd586_2805	Dda3937_00483	–
*Xanthomonas* campestris avirulence protein												
*svx*	BCS7_04170	29	9	15	3	10	6	8	4	Dd586_2807	Dda3937_00480	–
Citrate transporter												
*citN*	BCS7_06975	29	9	15	3	10	6	8	4	–	–	–
*sirB* locus												
*sirB1*	BCS7_10775	29	9	15	3	10	6	8	4	Dd586_2024	Dda3937_00039	Dd703_1858
*sirB2*	BCS7_10780	29	9	15	3	10	6	8	4	–	–	–
The 3-hydroxy-2-butanone pathway												
*budR/budA*	BCS7_03160/BCS7_03165	29	9	15	3	10	6	8	4	Dd586_0623/Dd586_0624	Dda3937_02312/Dda3937_02311	Dd703_3319/Dd703_3318
*budB*	BCS7_03170	28	9	15	3	10	6	8	4	Dd586_0625	Dda3937_02310	Dd703_3317
*budC*	BCS7_01530	29	9	15	3	10	6	8	4	Dd586_3192	Dda3937_03162	Dd703_0990
Unknown proteases												
-	BCS7_01920-BCS7_01935	6	0	15	3	10	0	4	0	Dd586_0366-Dd586_0369	–	Dd703_3577-Dd703_3574
-	BASYS00142	21	5	0	0	10	0	8	3	–	–	–
*yedU*	BASYS03033	29	9	15	3	9	0	8	4	Dd586_2787	Dda3937_02122	Dd703_0349
Regulation network												
Rsm system–post-transcriptional gene regulation												
*rsmA*	BCS7_16095	29	9	15	3	10	6	8	4	Dd586_3183	Dda3937_03151	Dd703_0999
*rsmB*^c^	–	29	9	15	3	10	6	8	4	1	1	0
*rsmC*	BCS7_03480	29	9	15	3	10	6	7	4	Dd586_0685	Dda3937_02456	Dd703_3258
ExpI-ExpR Quorum-Sensing system												
*expI/expR*	BCS7_00320/BCS7_00325	29	9	15	3	10	6	8	4	Dd586_0112/Dd586_0111	Dda3937_03219/Dda3937_03218	–
KdgR repressor												
*kdgR*	BCS7_09750	29	9	15	3	10	6	8	4	Dd586_1941	Dda3937_03685	Dd703_2055
RdgA-RdgB regulate system of pectin lyase production												
*rdgA/rdgB*	BCS7_09700/BCS7_09695	29	9	15	3	10	6	8	4	–	–	–
RNA polymerase sigma factor												
*rpoS*	BCS7_16895	29	9	15	3	9	6	8	4	Dd586_3302	Dda3937_03911	Dd703_0872
HexA repressor of extracellular enzyme and motility												
*hexA*	BCS7_14160	29	9	15	3	10	6	8	4	Dd586_2749	Dda3937_00364	Dd703_2551
Response regulator												
*expM*	BCS7_10215	29	9	15	3	10	6	8	4	Dd586_2148	–	Dd703_1959
ExpS-ExpA two-component system												
*expA*	BCS7_13455	29	9	15	3	10	6	8	4	Dd586_1482	Dda3937_03960	Dd703_1450
*expS*	BCS7_17085	29	9	15	3	9	6	8	4	Dd586_3330	Dda3937_00193	Dd703_0847
AepA catalytic protein												
*aepA*	ECA1022	15	7	0	0	10	6	5	1	–	–	–
Flagellar regulon transcriptional activators												
*flhD/flhC*	BCS7_13105/BCS7_13110	29	9	14	3	10	6	8	4	Dd586_1499/Dd586_1498	Dda3937_02784/Dda3937_02785	Dd703_1509/Dd703_1508

The details of these virulence related genes/ RNA fragment in each strain were shown in the Additional file 1: Table S6 and Table S6–1

*n* number of genomes tested. Numbers indicate the number of genomes that tested positive, *Pcc P. carotovorum* subsp. *carotovorum*, *Pcb P. carotovorum* subsp. *brasiliense*, *Pco P. carotovorum* subsp. *odoriferum*, *Pca P. carotovorum* subsp. *actinidiae*, *Ppo P. polaris*, *Pp P. parmentieri*, *Pa*, *P. atrosepticum*, *CPm Candidatus* P. maceratum

^a^Reference strains: part of data adapted from Zhou et al. (2015); ^b^*waaF*: W5S_4521, W5S_4522; ^c^*rsmB*, encoding a fragment of RNA acting as a global regulator of virulence, has no gene ID

At the subspecies level, *Pco* exhibited distinctly different gene composition when compared with other closely subspecies *Pca*, *Pcb* and *Pcc*. All *Pco* strains have the large DNA fragment (BCS7_01135-BCS7_01230) that contains several genes (*pli*, *cpa*, *tad*, and etc.) known to be involved in pili biosynthesis; this fragment is not present in other *P. carotovorum* strains with the exception of the *Pcb* strain BZA12. The *nosA* gene which encoded the out-membrane protein involved in the transport of metals [30] was absent in *Pcb* and *Pcc* while was present in *Pco* and *Pca*. Similarly, the *Pco* strains lack several genes carried by other subspecies strains, such as the major part of the pyelonephritis-associated pili (*pap*) operon (*papACD*; BASYS00091-BASYS00089), which are involved in the formation of adhesive pili in pathogenic bacteria [31, 32]. We also identified intermittent distribution of AepA exoenzyme regulatory protein among *Pcc* and *Pcb* strains, but not at all in *Pco* and *Pca* strains. The AepA protein may enable *Pectobacterium* to use atypical nitrogen sources, which are secreted by the host plant or microorganisms that colonize the host plant’s rhizosphere [33]. Many other genetic variations of pathogenicity related genes among these soft rot pectobacteria were shown in Table 2 and Additional file 1: Table S6. The findings of these unique genes confer to different species/subspecies provided a foundation for further work in elucidation of the genetic basis for pathogenicity in *Pectobacterium.*

#### Three T1SS protein secretion systems (has, prt, and lap) in Pectobacterium

The five protein secretion system types (T1SS-T4SS, and T6SS) found in 84 *Pectobacterium* genomes. Although all six secretion systems were reported to be present in *Pectobacterium* [11, 21] by Bell et al. and Glasner et al., improvements to annotation algorithms have resulted in the exclusion of T5SS in our study. The TNSSs of 3 *Dickeya* genomes used as the out-group also exhibited high diversity and were also discussed below.

**Fig. 5:**
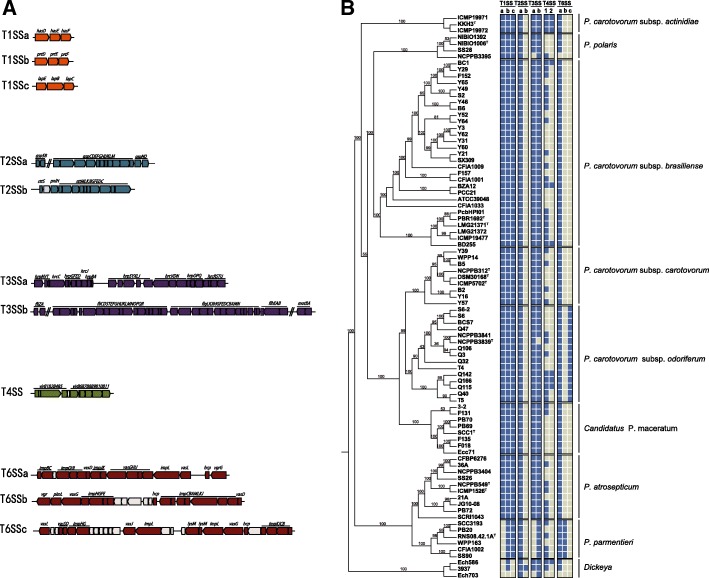
Gene clusters for TNSSs identified in the 84 *Pectobacterium* genomes. **a** Physical maps of Type I, II, III, IV, and VI secretion systems. Red colored arrows indicate known genes involved in the protein secretion systems. **b** Map showing the presence (blue feature) or absence (grey feature) of each type of secretion systems gene cluster, with a maximum likelihood phylogenetic tree based on the core genes of all the tested strains. a-c represents different subtypes of a given secretion system gene cluster; 1–2 represent the number of the copies of Type IV secretion system gene clusters

Three T1SSs (T1SSa, T1SSb, and T1SSc) were identified among the *Pectobacterium* strains (Fig. 5a and Additional file 1: Table S7). While T1SSb (*prtDEF*) and T1SSc (*lapBCE*) were present in all of the *Pectobacterium* strains in this study, T1SSa (*hasDEF*) was lost in *P. parmentieri*. Only T1SSb was found in the *Dickeya* strains we examined, of which *D. paradisiaca* Ech703 lacked the T1SSs. T1SSs secrete proteases and translocate molecules such as ions and toxins from the cytoplasm to the extracellular space [34], serving a range of functions. T1SSa of *Serratia marcescens*, for example, can translocate ions and secrete hemophores such as an extracellular heme-binding protein (HasA) to facilitate iron acquisition [35]. In *Pectobacterium*, T1SSb has been shown to secrete several Prt metalloproteases that are involved in attacking plant cell walls [36], and the repeats-in-toxin (RTX) protease PrtW mutants of *P. parmentieri* SCC3193 have altered virulence, suggesting a role for T1SSb in plant pathogenesis [37].

#### Species- and subspecies-specific features of T2SS

In contrast to the various T1SSs in *Pectobacterium*, only one T2SS cluster (T2SSa) was found among the *Pectobacterium* strains (Fig. 5b). However, the *P. parmentieri* strains and three *Dickeya* strains carried a variant T2SSa (Gsp) lacking *gspN* compared with that of other *Pectobacterium* strains. GspN is predicted to have an amino-terminal transmembrane helix, but its function has not yet been determined [38]. We also found that the length of *gspG* was 441 nt in all of *P. atrosepticum* and *P. parmentieri* strains and 456 nt in *Candidatus* P. maceratum, but found that this varied among the *P. carotovorum* strains. Moreover, the intergenic sequence length between *gspN* and *gspO* was 89 nt in all *Pco* and *Pcc* strains, but was 108 nt in all *Pcb* strains and 88 nt in all *Pca* strains. This intergenic length was not only specific to each subspecies, but was also specific at the species level, as the intergenic sequence length between *gspN* and *gspO* was 63 nt, 123 nt, and 89 nt, respectively, for each of the *P. parmentieri*, *P. atrosepticum*, and *Candidatus* P. maceratum strains. The extra T2SSb (Stt) presented in *D. dadantii* 3937 was not present in any tested *Pectobacterium* strains or in the other two *Dickeya* strains. T2SSb is known to encode a pectate lyase PnlH at the outer face of the outer membrane [39]. T2SS is extremely important for soft rot pathogens because it is responsible for the secretion of the major determinants of pathogenicity for this disease: the pectinases and cellulases that contribute to the characteristic rotting symptoms. It also secretes other factors related to pathogenicity, such as Svx and iron uptake proteins [40]. The inactivation of this secretory system renders *Pectobacterium* spp. avirulent [41].

#### T3SSa is absent in Pectobacterium parmentieri but found in other species

T3SSs include the virulence-associated Type III (T3SSa) and the flagellar secretion system (T3SSb). T3SSa clusters were absent from *P. parmentieri*, but were present in all of the strains of *P. carotovorum*, *Candidatus* P. maceratum, and *P. atrosepticum* (Fig. 5b and Additional file 1: Table S7). These results were in agreement with previous studies that failed to detect T3SSa or any of its surrounding genes in *P. parmentieri* SCC3193, a strain known to be virulent [22]. It is likely that T3SSa is not strictly required for the virulence and survival of *P. parmentieri*, although this cluster has been reported to contribute modestly to the virulence of *P. atrosepticum* and *P. carotovorum* [42]. A previous study reported some naturally T3SS-deficient *P. carotovorum* strains [43]; however, one *P. polaris* strain NCPPB 3395 from the Netherlands, previously assigned to *Pcc* [9], were identified as a T3SSa-deficient strain in the present study. T3SSa was present in *D. zeae* Ech586 and *D. dadantii* 3937, but this was lost in the *D. paradisiaca* Ech703. Whereas T3SSa had a species-specific distribution, T3SSb (BASYS02177-BASYS02229), which encodes important flagella components [44], was highly conserved among these *Pectobacterium* spp. and three *Dickeya* genomes except one *Pco* strain (NCPPB 3839^T^).

#### Irregular distribution of T4SS in Pectobacterium

T4SS is a versatile secretion system that can function in bacterial conjugation, DNA uptake/release, and cargo translocation. Several of the proteins encoded by the T4SS form three major sub-complexes: a cytoplasmic-inner membrane complex (VirB4 and VirB11), a core complex (VirB7, VirB9, and Vir10), and an extracellular pilus-associated complex (VirB2 and VirB5) (Fig. 5a). *P. atrosepticum* SCRI1043 VirB4 mutants are reported to exhibit reduced pathogenicity [11]. Distribution of T4SS was sporadic among *Pectobacterium* genomes and the T4SS-deficient strains were present among all five *Pectobacterium* species (*P. carotovorum*, *P. polaris*, *P. atrosepticum*, *P. parmentieri*, Candidatus *P. maceratum*) as well as *D. paradisiaca* Ech703 (Fig. 5b). The copy number also varied among the strains that carried it. For example, two copies of T4SS (BASYS00894-BASYS00885 and BASYS00696-BASYS00686), were identified in three *Pcb* strains (BC1, BZA12, and BD255), three *Pco* strains (Q115, Q142, and Q166), three *Pca* strains (ICMP 19972, ICMP 19971, and KKH3), as well as *D. zeae* Ech586, while the other strains only possessed a single copy.

#### A novel T6SS type (T6SSc) present in Pco strains

**Fig. 6:**
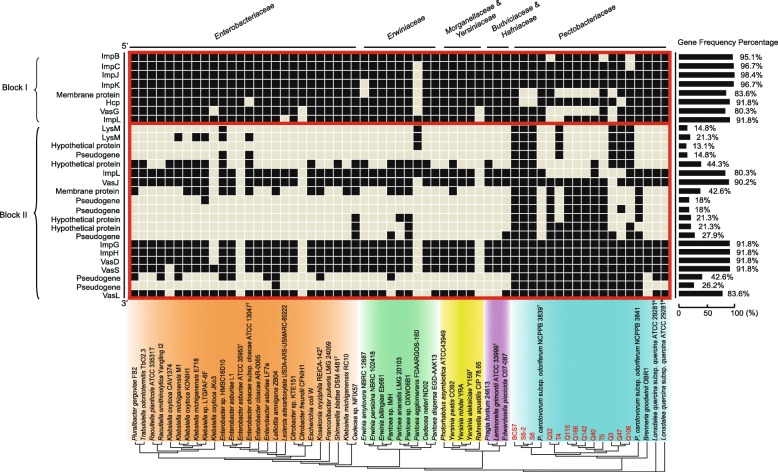
Genes of T6SSc presence in the 61 *Enterobacterales* genomes. A total of 61 of the 203 *Enterobacterales* genomes possessed the T6SSc cluster. The strains with red letters were from the newly sequenced genomes in this study; the others strains were from NCBI. The 28 genes of T6SSc of the BCS7 strain was used as a standard; we grouped the genes into two Blocks (Block I includes the first 8 genes (starting from the 5′ end) of the cluster; Block II includes the 20 terminal genes of the cluster at the 3′ end). Map showing the presence (black feature) or absence (grey feature) of each gene of the T6SSc cluster, with a maximum likelihood phylogenetic tree based on the core genes of 61 *Enterobacterales* genomes. Colored arrows indicate five clusters that divide these strains into different known families (*Enterobacteriaceae*, *Erwiniaceae, Morganellaceae* & *Yersiniaceae*, *Budviciaceae* & *Hafniaceae*, and *Pectobacteriaceae*) of the *Enterobacterales* order. a and b: two different sequenced genomes (accession numbers: NZ_JIBO00000000 and NZ_FNQS00000000) of the *Lonsdalea quercina* subsp. *quercina* strain ATCC 29281^T^, respectively. The frequency percentage of each gene of T6SSc among these genomes is shown on the right side next to the physical map

Our analysis of 84 *Pectobacterium* genomes identified three types of T6SSs (T6SSa-c), of which T6SSc is a novel type that identified and termed in this study (Fig. 5a and Additional file 1: Table S7). T6SSa was absent in *P. polaris* NCPPB 3395, but present in the rest of the *P. polaris* strains as well as the other four species (*P. atrosepticum*, *P. parmentieri*, *P. polaris*, *Candidatus* P. maceratum, and *P. carotovorum*). Similar to T1SS, T3SS, and T4SS, we found that the T6SS gene cluster was not present in the *D. paradisiaca* Ech703 genome, a finding consistent with results from a study by Zhou et al. [45]. T6SSb was a specific cluster that was only occurred in (all of the) *P. parmentieri* strains. Interestingly, we also found a novel T6SS that we termed T6SSc (BCS714475-BCS714610) which was only present in the *Pco* strains; the distribution of each of the genes of the T6SSc cluster varied among the *Pco* strains (Fig. 6 and Additional file 1: Table S8): nine genes were present in all 15 *Pco* strains, while the remaining 19 individual genes were variously present among the strains we surveyed. Similarity analysis based on DNA sequences revealed high genetic variation in pairwise identity comparisons of T6SSc between strains, ranging from 40.1% for the most divergent T6SSc of strains to 100% similarity for the most conserved.

### An expanded examination of the novel T6SSc and the *srl* operon

Based on the results of our comparative genomics study of *P. carotovorum* subspecies and the other four closely related species of *Pectobacterium*, we found that the novel cluster T6SSc and the *srl* operon were specific to *Pco* subspecies. To evaluate the distribution of this operon and T6SSc in other closely related strains, we conducted an expanded genome (203 genomes, Additional file 1: Table S8-S9) analysis with all available genomes data of *Pectobacterium* downloaded from GenBank in July 2018 as well as other sequences retrieved from GenBank for representative strains from each branch of the phylogenetic trees of the *Enterobacterales* order from a study by Adeolu et al. [46].

#### The highly conserved srl operon in Pco subspecies

A total of 203 genomes of *Enterobacterales* strains were analyzed: only 66 of them possessed this *srl* operon (Additional file 1: Table S9). In addition to all of the sequenced *Pco* strains (including 13 from this study and the 2 from NCBI), we did not find any other *Pectobacterium* spp. or *Dickeya* spp. which cause similar soft rot diseases that harbored this operon. Besides the *Pco* strains, the *srl* operon was also present in two sequenced *Brenneria* strains and in one of the three sequenced *Sodalis* strains of the *Pectobacteriaceae* family. The *srl* operon occurred among most genomes of the *Enterobacteriaceae* (81.1%), *Yersiniaceae* (80.0%), and *Erwiniaceae* (62.5%) families, but is not present in the *Morganellaceae*, *Budviciaceae*, or *Hafniaceae* families. We found some plant pathogens with a broad host range (e.g., *Pantoea ananatis*) [47], some that infect hosts containing high levels of sorbitol (e.g., *Erwinia* spp.) [48–50], and some growth-promoting rhizobacteria (e.g., nitrogen-fixing strain) [51] also possess this operon. In addition, we aligned the *srl* operon sequences (protein and DNA) from these 66 strains and conducted phylogenetic and similarity (MUSCLE) analyses. The similarity analysis (protein and DNA) revealed few genetic differences (pairwise similarity > 99.4% in protein sequences; pairwise similarity > 99.5% in DNA sequences) in the *srl* operon between different *Pco* strains. Phylogenetic analysis based on DNA and protein sequences of the *srl* operon (Additional file 3: Figure S2) both showed that *Pco* strains were clustered into the clade with *Erwinia* spp., but separated from the clades containing *Enterobacteriaceae* spp. and *Yersiniaceae* spp. This result was consistent with the *srl* operon of *Pco* strains sharing highest similarity with those of *Erwinia* spp. (69.1–71.3% in DNA sequence and 78.6–80.0% in protein sequence).

#### Genetic variation of the novel secretion system T6SSc

Sixty-one Of the 203 *Enterobacterales* genomes we examined possessed the T6SSc cluster. In contrast to the high conservation of the *srl* operon among *Pco* strains, relatively high genetic variation was observed among the *Pco* T6SSc cluster sequences. Despite of this, the phylogenetic trees (not shown) built based on their T6SSc cluster sequences (protein and DNA) clustered all of the 15 *Pco* strains sequenced to date into a clade which was clearly separated from the other *Enterobacterales* strains. The T6SSc cluster was also present in a limited number of *Brenneria goodwinii* and *Lonsdalea quercina* strains in the *Pectobacteriaceae*, as well as being present sporadically among the other 6 families of the *Enterobacterales* order (Additional file 1: Table S8).

An analysis that screened for the presence/absence of individual genes of the T6SSc cluster in 61 *Enterobacterales* genomes showed two clear distribution patterns (Fig. 6). It suggested that the cluster consisted of two blocks of genes: the first, Block I, includes the first 8 genes (starting from the 5′ end) of the cluster, while Block II includes the 20 terminal genes of the cluster at the 3′ end. We found that genes of Block I were more highly conserved than those of Block II, a finding that merits further study that may help to clarify the evolutionary history of *Pco* species within the *Pectobacterium* genus.

## Discussion

Historical assessments which assert that *Pcc* has the widest host range among the *Pectobacterium* taxa are due for revision, considering that multiple studies which examined the pathogenicity and host ranges of *Pcb* and *Pco* have now established a broad spectrum of hosts for these subspecies as well [5, 52, 53]. Many strains previously known as *Pcc* have also been re-classified into other species in recent years [8, 9]. Our finding that the *P. peruviense*, *P. polaris*, and *Candidatus* P. maceratum strains did not possess either the T6SSc cluster or the *srl* operon, viewed alongside our finding that no *Pcc* strains harbored either cluster, are consistent with the recently proposed adjustments to the classification of *Pectobacterium* species, which have posited that the newly proposed species are derived from *Pcc*. We also noticed that the genomic traits of *Pca* for some pathogenicity-related genes as well as the CRISPR-Cas systems, were not consistent with any of the three-known *Pcc*, *Pcb* and *Pco* subspecies, which is further support to the new subspecies proposal.

Interestingly, an *srl* operon related to sorbitol metabolism was specific to *Pco* genomes among the *Pectobacterium* genus based on the BLAST analysis using both DNA and amino acid sequences. Other studies have reported that mutants with loss-of-function for this operon in another phytopathogen (*Erwinia amylovora*) with a sorbitol deficiency were unable to grow on minimal medium with sorbitol [26]. Our biochemical test results also confirmed that these *Pco* strains with the *srl* operon could produce acid from sorbitol or could utilize this carbon source, while other strains lacking this operon like *Pcc* and *Pcb* could not. This is further supported by some previous studies which showed that *Pectobacterium* strains and the type strains of other species, such as *Pcc*, *P. wasabiae*, *P. betavasculorum*, and *P. atrosepticum* could not utilize/produce acid from sorbitol but that the type *Pco* strain as well as other *Pco* strains could [54–57]. However, the ability of these *Pectobacterium* type strains to use sorbitol is still obscure; there are conflicting reports about the biochemical phenotypes for these strains reported in various studies [8, 52, 58].

Moreover, although the sequences of the *srl* operon from *Pco* strains are conserved, and are closely related to those present in *Erwinia* spp., whether the *Pco* strains acquired this operon from *Erwinia* spp., or if they acquired it from a common ancestor (and may have subsequently lost it in certain strains) still awaits clarification. Further, although this operon is strictly required for the pathogenicity of *E. amylovora* [26], its function in *Pco* is still unknown; it is clearly important considering its high level of conservation in *Pco* strains.

The other specific cluster to *Pco* subspecies among *Pectobacterium* is T6SSc; while the T6SSa and T6SSb type clusters have been reported extensively, we are unaware of other studies reporting a cluster with the gene order we detected in this T6SSc type cluster. T6SS have been shown to function in host cell adherence and in competition with other microbes, but the role of this class of gene cluster in *Pectobacterium* during infection remains unclear [2]. Our results confirmed the previously reported finding that *P. atrosepticum* contains only one T6SS (T6SSa), whereas *P. parmentieri* harbors two such machineries (T6SSa and T6SSb) [22]*.* However, in contrast to the previous description that *P. carotovorum* stains only have one T6SS (T6SSa), we detected T6SSc in these species. Although the components of this system were not strictly consistent among the *Pco* strains, and its function and potential targets must of course be further determined, it will be interesting to explore the possible functional contribution of this novel T6SSc to the pathophysiology of soft rot diseases. Many other genetic variations including pathogenicity-related genes as well as CRISPR-Cas systems that were revealed among the subspecies and species by this study lay a foundation for future work to elucidate the genetic basis of pathogenicity in *Pectobacterium.* It will also be interesting to determine which among these genomic variations in the different species/subspecies contribute to host ranges and geographic distributions.

## Conclusion

Our findings significantly expand the body of knowledge surrounding the genetic basis for pathogenicity of soft rot bacteria in general. The genetic variations catalogued in this study also provide new insights into the evolutionary history of *Pectobacterium* and give extensive definitive evidence for accurately classifying members of the *Pectobacterium* species and subspecies. These analyses will provide a foundation for generating hypotheses about pathogenicity, host-bacteria interactions, taxonomy, and phylogenetic relationships among soft rot bacteria and other phytopathogens.

## Methods

### Collection and phenotyping of 32 Chinese *Pectobacterium carotovorum* isolates

Thirty-two strains from 3 economically important Chinese vegetables, Chinese cabbage (*Brassica rapa subsp. pekinensis*), bok choy (*Brassica rapa subsp. chinensis*), and celery (*Apium graveolens*) were collected from North China (Beijing area). Typical symptoms of soft rot were observed on the petioles and stems of these plants, which were grown in cultivated field in different districts of Beijing (Additional file 1: Table S3). Isolation of bacteria from diseased samples was carried out according to previous studies [54], were identified by Yu Tian and Xiaoying Li, previously, and were preserved in Beijing Key Laboratory of Agricultural Genetic Resources and Biotechnology, Beijing Academy of Agriculture and Forestry Science. These strains were tested for pathogenicity by inserting one pipette tip containing 10 μL of bacterial suspension (2 × 10^8^ CFU/mL) 2 mm into each sterilized Chinese cabbage petioles surface with sterile distilled water as control. The petioles were then incubated at 28 °C and 95% relative humidity for 24 h before the diameter of the rotting tissue in each petiole was measured. Each strain was represented by three replicate petioles, and the experiment was performed three times. The data were subjected to one-way ANOVAs using SPSS 10.0. Means were compared by the Duncan’s tests, and statistical significance was determined at 5% levels. Strains were re-isolated successfully from symptomatic Chinese cabbages to complete Koch’s postulates. All strains were assayed with several biochemical tests: reducing substances from sucrose; acid production from D-sorbitol, D-arabitol, palatinose, and α-methylglucoside; utilization of citrate, and growth in 5% NaCl and at 37 °C [59]. Moreover, the 94 phenotypes (the oxidation of the 71 carbon sources and chemical susceptibility to 23 compounds) were analyzed using GEN III OmniLog system by the BIOLOG™ GEN III microplates [60].

### Genome sequencing, assembly, and annotation of the 32 newly isolated *Pectobacterium carotovorum* strains

Genomic DNA was extracted and purified using an EasyPure Genomic DNA Kit (Transgen, China) according to the product instructions. Sequencing of the *Pcb* BC1 genome (accession: CP009769) was performed using the Illumina Hiseq 2000 platform with 2 Kb mate pairs and 200 to 500 bp unique paired-ends insert size libraries. The BC1 sequence was assembled by SOAPdenovo 2.0 software representing a 335-fold average genomic coverage. The scaffold order was based on the PCC21 reference genome, and a PCR-based genomic walking method was used to fill gaps between scaffolds. Sequencing of the *Pco* BCS7 genome (accession: CP009678) was performed with Pacific Biosciences RS II sequencing technology. A 10 Kb Single-Molecule Real-Time (SMRT) library was prepared from sheared genomic DNA using a 10-kb template library preparation workflow. The library was sequenced on 2 SMRT cells, providing about 137-fold genome coverage. The denovo assembly of the BCS7 reads was performed with the Hierarchical Genome Assembly Process (HGAP) algorithm in SMRT Portal (version 2.3.0). The genomes of the additional 30 *P. carotovorum* strains were sequenced with MPS (massively parallel sequencing) Illumina technology. A paired-end DNA library with an insert size of 500 bp was constructed and was sequenced using an Illumina HiSeq. Quality control of paired-end reads was performed using an in-house program to filter the low-quality reads, Illumina PCR adapter reads, and duplication reads. After filtering, all 30 of the strains had the sequencing coverage between 65-fold and 200-fold. Denovo assembly of the reads was performed with the SOAPdenovo algorithm (http://soap.genomics.org.cn/soapdenovo.html). All reads were used for further gap closure.

Coding genes of these 32 newly sequenced strains were predicted with GeneMark software by the self-training program GeneMarkS (version: 4.6b, http://topaz.gatech.edu/GeneMark/). Transfer RNA (tRNA) genes were predicted with tRNAscan-SE (version: 1.3.1, http://gtrnadb.ucsc.edu/) [61], Ribosome RNA (rRNA) genes were predicted with rRNAmmer [62], and sRNAs were predicted by BLAST against the Rfam database. Long terminal repeats sequences of all the tested genomes in this study were predicted using Repeat Masker (http://www.repeatmasker.org/) and tandem repeats were analyzed using Tandem Repeat Finder (http://tandem.bu.edu/trf/trf.html). Among these tandem repeats, arrays with repeat units 15 bp to 65 bp will be considered as minisatellites DNA. Gene functional annotations of each of the 32 genomes was performed by BLAST analysis against multiple databases, including NCBI NR (Version: 20150405, http://www.ncbi.nlm.nih.gov/), KEGG (Version: 59, http://www.genome.jp/kegg/), COG (Version: 20090331, http://www.ncbi.nlm.nih.gov/COG/), GO (Version: 20150405, http://geneontology.org/), Swiss-Prot (Version: 20150414, http://www.ebi.ac.uk/uniprot/), PHI (Version: 3.6, http://www.phi-base.org/), VFDB (Version: Tue May 5 10:06:01 2015, http://www.mgc.ac.cn/VFs/main.htm), MvirDB database [63], ARDB (Version: 1.1, http://ardb.cbcb.umd.edu/), CARD (Version: 20140415, http://arpcard.mcmaster.ca/), CAZy (Version: 20141020, http://www.cazy.org/), TCDB (Version: 20110715, http://www.tcdb.org/). For each BLAST hit (a given ORF), the best alignment with the lowest e-value and highest score was retained; to be retained, a hit required at least 0.4 identity for a sequence at 40% of a given alignment’s length. All 32 genomes newly sequenced in this study have been deposited in the NCBI genome database (Accession numbers were shown in Additional file 1: Table S3).

### Comparative genomic analysis

A comprehensive comparative genomics study of 84 *Pectobacterium* genomes (including the 32 newly sequenced genomes in this study and 52 other *Pectobacterium* genomes published in GenBank by July 2018, for strains including *P. carotovorum*, *P. polaris*, *Candidatus* P. maceratum, *P. atrosepticum*, and *P. parmentieri*) was conducted using three complete *Dickeya* genomes (*D. zeae* Ech586, *D. paradisiaca* Ech703, and *D. dadantii* 3937) as the reference outgroup. Note that the previously reported *P. carotovorum* strain 3–2, as well as the *Pcc* strains ATCC 39048 and Ecc71, were reclassified in this study (Fig. 2a); therefore, strains Ecc71 and 3–2 were part of the *Candidatus* P. maceratum group and strain ATCC 39048 was part of the *Pcb* group in each of the bioinformatics analyses of this in this comparative genomics study. The verified classification and detailed information for each of the strains are shown in Additional file 1: Table S3. All of these 84 *Pectobacterium* genomes were used for the following bioinformatics analyses.

#### Whole-genome SNP detection and identity score analyses

Genome-Wide SNP detection was performed using MUMmer comparison software: the whole-genome sequence of BC1 (the reference) was compared with genome sequence of the other strains to identify differences. BLAT (BLAST-Like Alignment Tool) software was then used to compare the sequences adjacent to the SNP loci to verify a given SNP’s validity [64]. Repeat regions were detected by BLAST, Repeat Masker, and Tandem Repeats Finder software. If the length of the SNP adjacent sequence alignment in the BLAT results was less than 101 bp, or if a SNP was found in a repeat region, the data was considered as suspect and was removed. We performed a phylogenetic analysis based on SNP data: for all species, all SNPs were joined in the same order. PhyML software (Version: 3.0, http://www.atgc-montpellier.fr/phyml/) was then used to construct the phylogenetic tree with a maximum likelihood model. The number of bootstrap replications was set to 1000.

To identify genetic relatedness in any two strains, pairwise Identity Scores (IS) were calculated [65]. The IS is the ratio of unique SNPs between two accessions; this was determined using an in-house Python script and visualized with a heat map which produced with the ggplot2R package.

#### Pan-genome analyses

Eighty-four *Pectobacterium* genomes, including 56 *P. carotovorum*, 6 *P. parmentieri*, 10 *P. atrosepticum*, 8 *Candidatus* P. maceratum, and 4 *P. polaris* genomes were used in the pan-genome analysis of four closely related species of the *Pectobacterium* genus (Additional file 1: Table S3). This included the 9 *Pcc*, 29 *Pcb*, 15 *Pco*, and 3 *Pca* genomes that were used in the aforementioned pan-genome analysis of the *P. carotovorum* species. Subspecies core orthologous genes and strain-specific unique genes were also examined in the 9 *Pcc*, 15 *Pco*, 29 *Pcb*, and 3 *Pca* genomes sequences, respectively. Specifically, it should be noted that the five pan-genomes of each species (that is, the pan-genome for 56 *P. carotovorum* genomes*,* that of the 6 *P. parmentieri*, 10 *P. atrosepticum*, 8 *Candidatus* P. maceratum, and 4 *P. polaris* genomes, respectively) were used as the petals for generating the Venn diagram for the *Pectobacterium* genus.

Likewise, the pan-genomes for each of the four subspecies (that is 29 *Pcb,* 3 *Pcc*, 15 *Pco,* and 3 *Pca* genomes, respectively) were used as the petals to generate the Venn diagram for the *P. carotovorum* species. The core and pan genes identified and clustered using CD-HIT rapid clustering of similar proteins software [66, 67], with a threshold of 70% pairwise identity and a 0.6 length difference cut-off in amino acid sequences. Genes that did not reach the threshold were used to initiate a new cluster. Genes from multiple species in each core gene cluster were aligned to each other to make a multi-sequence alignment using MUSCLE (Version: 3.8.31, http://www.drive5.com/muscle). The CD-HIT analysis also identified the genes that were unique to each species/subspecies of *Pectobacterium*, and their functions differences based on COG annotations were performed. Finally, a core-gene phylogenetic tree of these *Pectobacterium* strains was constructed with three *Dickeya* strains as the outgroup. The PhyML (Version: 3.0, http://www.atgc-montpellier.fr/phyml/) software was used with a maximum likelihood model. The number of bootstrap replications was set to 1000.

#### Gene cluster analysis of orthologs

BLAST analysis using both DNA and amino acid sequences was used to identify the presence of homologs of previously known genes (e.g., pathogenicity-related genes) in the genomes in this study. For each BLAST hit (a given ORF), the best alignment with the lowest e-value and highest score was retained; to be retained, a hit required at least a 0.4 identity for a sequence at 40% of a given alignment’s length. Detection of CRISPR-Cas system components. The CRISPR Finder online tool (http://crispr.i2bc.paris-saclay.fr/Server/) was used to identify all of the predicted CRISPR components for each strain. Predicted *cas* genes were also identified with BLAST analysis according to Makarova (2011) [68]. The CRISPR and *cas* results were combined to determine the predicted locations of the CRISPR-Cas system(s) in each strain.

### An expanded examination of the novel T6SSc and the srl operon

After confirming the *srl* operon and T6SSc were two *Pco*-specific clusters within 84 *Pectobacterium* and 3 reference *Dickeya* genome sequences tested above, we examined its distribution in the *Pectobacterium* genus and even in the order *Enterobacterales*. This analysis included the 203 genomes, including all of the available *Pectobacterium* downloaded from GenBank in July 2018, as well as other sequences from GenBank for representative strains from each branch of the phylogenetic trees of *Enterobacterales* as reported by Adeolu et al. [46]. These 203 *Enterobacterales* genomes covered 62 species of 31 genera across 7 families (*Enterobacteriaceae*, *Erwiniaceae, Morganellaceae*, *Yersiniaceae*, *Budviciaceae*, *Hafniaceae*, and *Pectobacteriaceae*) of the *Enterobacterales* order. BLAST analysis using both DNA and amino acid sequences was used to identify the presence of homologs of the genes of the *srl* operon and T6SSc cluster in these 203 *Enterobacterales* genomes with the sequences of the BCS7 strain (the only *Pco* strain with a complete genome) used as a standard. After identifying homologs in these strains, we examined the similarity of the sequences for these two clusters using a maximum likelihood phylogenetic tree based on both the DNA and amino acid sequences. We also calculated the ratio of the similarity based on the total length of alignment of each couple strains in the matrix based on the results of multi-sequence alignment (both the DNA and amino acid sequences) using MUSCLE software.

## Additional files

Additional file 1: **Table S1.** The biochemical characters and pathogenicity of 32 Chinese *Pectobacterium carotovorum* strains. **Table S2.** Selected phenotypic characteristics of *Pectobacterium carotovorum* subsp. *brasiliense*, *P. carotovorum* subsp. *carotovorum* and *P. carotovorum* subsp. *odoriferum*. **Table S3.** Eighty-four *Pectobacterium* genomes used in this study, with accession number, host origin and location information. **Table S4.** The general genomic features of the 84 *Pectobacterium* genomes. **Table S5.** The CRISPR-Cas systems identified in the 84 *Pectobacterium* genomes. **Table S6.** Presence of genes encoding general virulence factors in the 84 *Pectobacterium* genomes. Table S6–1. The position of *rmsB*, the RNA fragment related to virulence in the 84 *Pectobacterium* genomes. **Table S7.** Gene clusters for TNSSs identified in the 84 *Pectobacterium* genomes. **Table S8.** Presence of the T6SSc gene cluster in the 203 *Enterobacterales* genomes. **Table S9.** Presence of the *srl* operon in the 203 *Enterobacterales* genomes. (XLSX 751 kb)

Additional file 2: **Figure S1.** Soft rot symptoms in *Brassica rapa subsp. pekinensis* (**a**), *Brassica rapa subsp. chinensis* (**b**), and *Apium graveolens* (**c**). (PDF 112808 kb)

Additional file 3: **Figure S2.** Phylogenetic tree based on the amino acid sequences of the *srl* operon in the 66 *Enterobacterales* strains. A total of 66 of the 203 *Enterobacterales* genomes were found to possess the *srl* operon in this study. The phylogenetic trees based on DNA and amino acid sequences were almost identical. The strains with red letters were the newly sequenced in this study. They were clustered into the clade containing the *Ewinia* strains. The tree was constructed using a maximum likelihood method and was generated with 1000 bootstrap replicates. (PDF 763 kb)

## Abbreviations

BLAT: BLAST-like alignment tool
COG: Clustering of orthologous groups
CRISPR-Cas: Clustered regularly interspaced palindromic repeats/CRISPR-associated proteins
ECA: Enterobacterial common antigen
IS: Identity scores
LPS: Lipopolysaccharide
Nip: Necrosis-inducing protein
Pap: Pyelonephritis-associated pili
*Pca*: *P. carotovorum* subsp. *actinidiae*
*Pcb*: *P. carotovorum* subsp. *brasiliense*
*Pcc*: *P. carotovorum* subsp. *carotovorum*
*Pco*: *P. carotovorum* subsp. *odoriferum*
PCWDEs: Plant cell wall-degrading enzymes
PGAAP: Prokaryotic Genome Annotation Pipeline
SNPs: Single nucleotide polymorphisms
Svx: A homologue of an avirulence protein of *Xanthomonas*
TNSS: Protein secretion system
