# Supplementary figures and images for: Comparative genomics of 84 Pectobacterium genomes reveals the variations related to a pathogenic lifestyle
Source: BMC Genomics. 2018 Dec 7;19:889. doi: 10.1186/s12864-018-5269-6 (PMC6286560; doi:10.1186/s12864-018-5269-6)

A

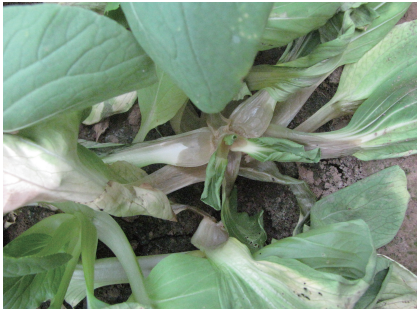

B

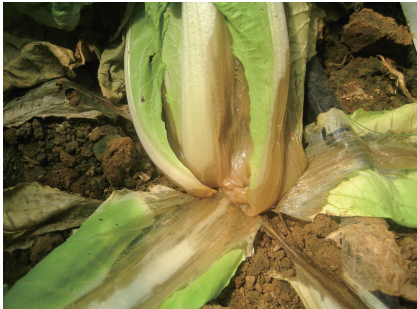

C

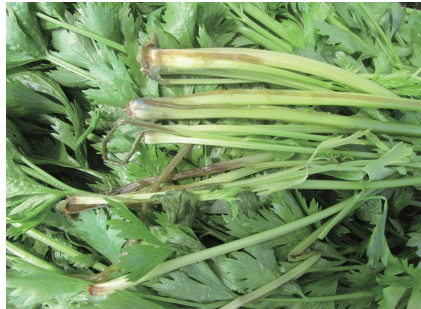

Supplement: Supplementary file 2 — Figure S1. Soft rot symptoms in Brassica rapa subsp. pekinensis (a), Brassica rapa subsp. chinensis (b), and Apium graveolens (c). (PDF 112808 kb) [file 12864_2018_5269_MOESM2_ESM.pdf]

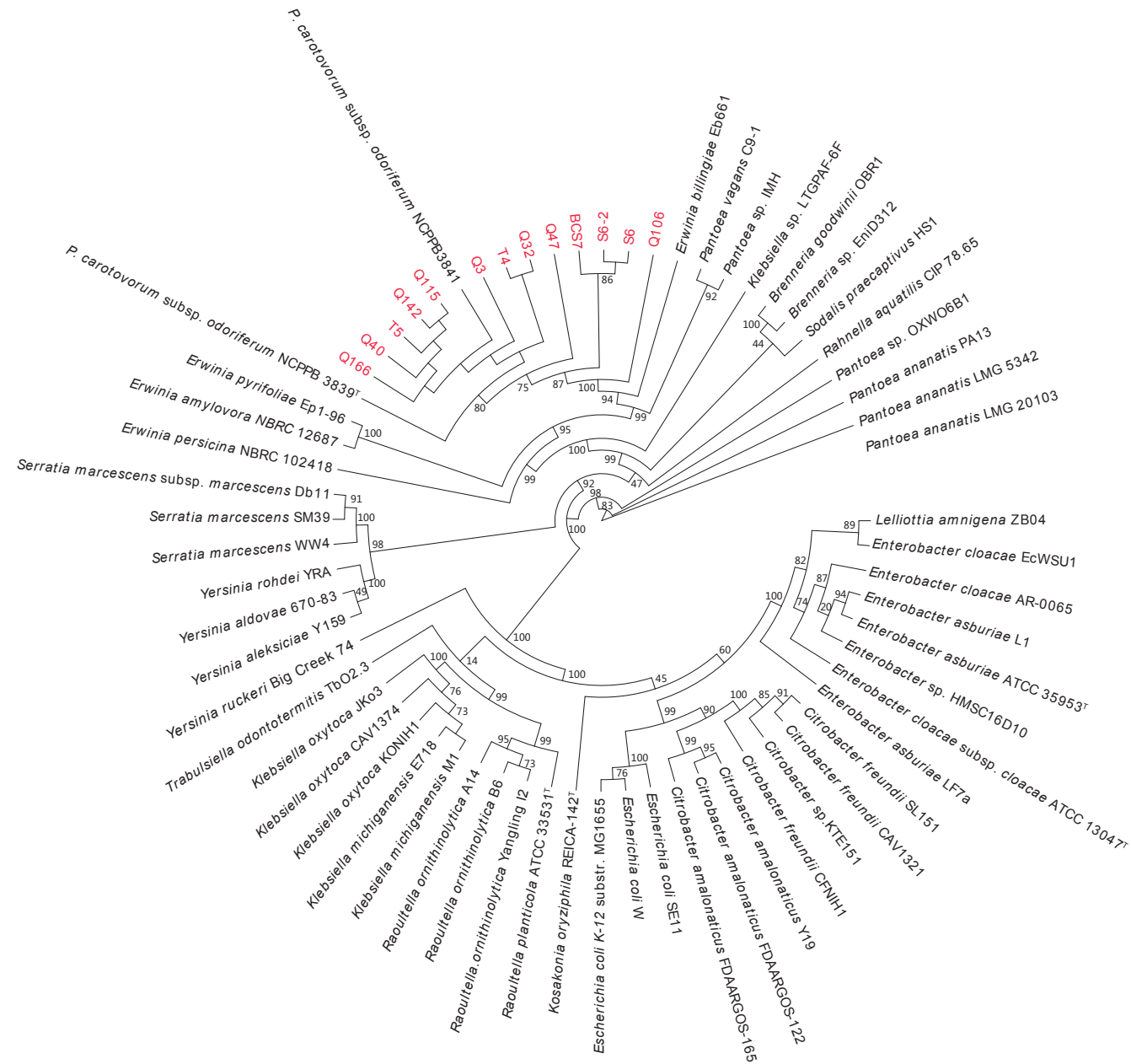

Supplement: Supplementary file 3 — Figure S2. Phylogenetic tree based on the amino acid sequences of the srl operon in the 66 Enterobacterales strains. A total of 66 of the 203 Enterobacterales genomes were found to possess the srl operon in this study. The phylogenetic trees based on DNA and amino acid sequences were almost identical. The strains with red letters were the newly sequenced in this study. They were clustered into the clade containing the Ewinia strains. The tree was constructed using a maximum likelihood method and was generated with 1000 bootstrap replicates. (PDF 763 kb) [file 12864_2018_5269_MOESM3_ESM.pdf]
